# Assessment of Seasonal Stochastic Local Models for Glucose Prediction without Meal Size Information under Free-Living Conditions

**DOI:** 10.3390/s22228682

**Published:** 2022-11-10

**Authors:** Francesco Prendin, José-Luis Díez, Simone Del Favero, Giovanni Sparacino, Andrea Facchinetti, Jorge Bondia

**Affiliations:** 1 Department of Information Engineering (DEI), University of Padova, Via G. Gradenigo 6/B, 35131 Padova, Italy; 2 Instituto Universitario de Automática e Informática Industrial, Universitat Politècnica de València, Camino de Vera, s/n, 46022 València, Spain; 3 Centro de Investigación Biomédica en Red de Diabetes y Enfermedades Metabólicas Asociadas (CIBERDEM), Instituto de Salud Carlos III, 28029 Madrid, Spain

**Keywords:** type 1 diabetes, glucose prediction, fuzzy clustering, seasonal local models

## Abstract

Accurate blood glucose (BG) forecasting is key in diabetes management, as it allows preventive actions to mitigate harmful hypoglycemic/hyperglycemic episodes. Considering the encouraging results obtained by seasonal stochastic models in proof-of-concept studies, this work assesses the methodology in two datasets (open-loop and closed-loop) recorded in free-living conditions. First, similar postprandial glycemic profiles are grouped together with fuzzy C-means clustering. Then, a seasonal stochastic model is identified for each cluster. Finally, real-time BG forecasting is performed by weighting each model’s prediction. The proposed methodology (named C-SARIMA) is compared to other linear and nonlinear black-box methods: autoregressive integrated moving average (ARIMA), its variant with input (ARIMAX), a feed-forward neural network (NN), and its modified version (NN-X) fed by BG, insulin, and carbohydrates (timing and dosing) information for several prediction horizons (PHs). In the open-loop dataset, C-SARIMA grants a median root-mean-squared error (RMSE) of 20.13 mg/dL (PH = 30) and 27.23 mg/dL (PH = 45), not significantly different from ARIMA and NN. Over a longer PH, C-SARIMA achieves an RMSE = 31.96 mg/dL (PH = 60) and RMSE = 33.91 mg/dL (PH = 75), significantly outperforming the ARIMA and NN, without significant differences from the ARIMAX for PH ≥ 45 and the NN-X for PH ≥ 60. Similar results hold on the closed-loop dataset: for PH = 30 and 45 min, the C-SARIMA achieves an RMSE = 21.63 mg/dL and RMSE = 29.67 mg/dL, not significantly different from the ARIMA and NN. On longer PH, the C-SARIMA outperforms the ARIMA for PH > 45 and the NN for PH > 60 without significant differences from the ARIMAX for PH ≥ 45. Although using less input information, the C-SARIMA achieves similar performance to other prediction methods such as the ARIMAX and NN-X and outperforming the CGM-only approaches on PH > 45min.

## 1. Introduction

Type 1 diabetes (T1D) is a chronic autoimmune disease that impairs insulin production. As a consequence, T1D individuals are required to maintain their blood glucose (BG) in a safe range (70–180 mg/dL) via insulin injections, carbohydrate (CHO) intake, and physical exercise to avoid the consequences of harmful events, known as hyperglycemia (BG > 180 mg/dL) and hypoglycemia (BG < 70 mg/dL). Mitigating the duration and the occurrence of these episodes is the main goal of the standard T1D therapy, which also requires frequently monitoring BG concentrations to correctly tune the amount of CHO and insulin boluses to administer along the day. In the last 15 years, continuous glucose monitoring (CGM) sensors have become a widely used tool for real-time BG monitoring in T1D management. These devices provide BG levels almost continuously (i.e., from 1 up to 5 min) for several days [1,2] and often embed visual and acoustic alerts when BG exceeds the normal glucose ranges. These devices have been proven to ease the daily routine burden of T1D individuals and to improve the control of BG inside the desired glucose range (70–180 mg/dL) [3]. However, CGM-based preventive alerts triggered before reaching critical levels would be even more helpful than detecting events already started. In fact, preventive warnings enable targeted measures to avoid or mitigate harmful episodes. The prediction of future BG levels enables several applications that can improve the management of T1D, for instance:

- In insulin pump systems, it can (and in some systems, it does [4]) trigger insulin delivery suspensions [5,6,7], if a hypoglycemic episode is predicted;
- In a decision support system (DSS)—a composite tool that implements multiple algorithms to support the patient in the decision-making process—glucose prediction can be used to suggest the correct amount of CHO to avoid low glucose values [8,9];
- In artificial pancreas systems (AP) [10,11], BG prediction uses by closed-loop control algorithms to automatically increase or decrease insulin delivery.

For these reasons, there have been several research efforts investigated BG prediction [12] in order to develop methodologies for an accurate prediction of the future BG concentrations. In particular, two main categories can be found: algorithms fed only by the past history of the CGM signal, such as [6,13,14,15], or fed by CGM data plus additional information such as insulin, CHO, or physical exercise, as in [16,17]. Moreover, as demonstrated in several comprehensive reviews about glucose prediction algorithms [12,18], the diabetes research community has intensively focused on developing black-box methodologies, using techniques developed in the field of time series forecasting, system identification, and machine and deep learning [19,20,21,22,23,24].

Among the possible approaches for glucose prediction, the use of stochastic seasonal models, as well as clustering techniques is still only partially explored in the literature. In fact, seasonal models were introduced for the first time in [25], and the combined use of seasonal models along with clustering techniques was introduced in [26,27]. In these works, the methodology was developed and validated only on well-controlled datasets: the first [26] was recorded during in-hospital clinical trials, while the second one [27] was obtained by exploiting the educational version of the UVA/Padova simulator [28]. In both cases, the results were encouraging since the proposed approach based on seasonal models and clustering outperformed all the state-of-the-art techniques for BG prediction. However, a real-time assessment on data recorded in free-living conditions is still needed. In fact, dealing with real data poses some issues about the completeness and reliability of stored information, which can degrade the ability of the algorithms to accurately forecast BG levels [16,18]. Moreover, glucose dynamics recorded in free-living conditions can be much more complex to describe than the ones obtained by simulations or others recorded during in-hospital trial sessions, since in the first case, the patient is exposed to substantially larger disturbances to glucose homeostasis.

The aim of this work is to fill this gap by providing an assessment of the clustering and seasonal local modeling methodology for glucose prediction proposed in [26,27] on two real datasets of different sizes (11 and 13 subjects monitored for 8 weeks and about 5 months, respectively) and obtained with different insulin dosing strategies (manual open-loop and closed-loop control). For each subject, CGM postprandial periods are grouped into clusters, and then, for each cluster, an optimal seasonal autoregressive integrated moving average (SARIMA) model is identified. Finally, the real-time BG forecasting is performed by weighting the prediction of each model. Considering several prediction horizons (PHs), the predictive performance of the proposed methodology (named C-SARIMA) is compared with that of different approaches: an individualized autoregressive integrated moving average (ARIMA) model and a feed-forward neural network (NN) based on CGM data only; an individualized autoregressive integrated moving average with exogenous inputs (ARIMAX) model and a variant of the NN, namely NN-X, fed by CGM, insulin, and CHO information (timing and amount). Notably, previous studies showed that the ARIMA is the best-performing linear algorithm for blood glucose forecasting using CGM data only [29], while its extension, ARIMAX, is one of the most-suitable options when additional information, such as insulin and CHO information, is available. Notably, both the ARIMA and ARIMAX models allow achieving accurate prediction performance even if compared to other nonlinear and more complex algorithms [16,29,30]. Our work demonstrates that, for PH > 45 min, the C-SARIMA outperforms individualized ARIMA models and there is no statistically significant difference when compared to individualized ARIMAX with the practical advantage of the minimal input information needed (i.e., meal timing).

## 2. Materials and Methods

### 2.1. Datasets

The first dataset used in this study is the Ohio Type 1 Diabetes Mellitus dataset [31], from now on referred to as OhioT1DM. The OhioT1DM dataset was updated in the 2020 release, and it comprises 12 subjects with T1D monitored for 8 weeks. The subjects wore a Medtronic Enlite CGM device (sampling time is 5 min) along with an insulin pump (Medtronic 530G or 630G) and a wearable system (Basis Peak fitness or Empatica Embrace) to measure physiological variables, for instance: skin temperature, skin conduction, and heart rate. Moreover, the dataset provides subjects self-reported information about meals: timing, amount and type (i.e., breakfast, lunch, dinner, snack, hypoglycemia treatment). Since self-reported mealtime is crucial information for the real-time validation purposes of this work, Subject ID 567, which did not record any meal during the last 10 days of monitoring, was discarded.

Each subject comprising the OhioT1DM dataset was split into a training set (about 82% of the entire monitoring period) consisting of the initial 6 weeks of monitoring and a test set (the remaining 18%) composed by the last 10 days.

The second dataset was collected in a multicenter clinical trial (www.clinicaltrial.gov: NCT02137512) aimed to assess the long-term use of a hybrid closed-loop insulin delivery system developed at the University of Virginia [32]. From now on, it will be referred to as the CTR3 dataset. The CTR3 dataset comprises 14 individuals with T1D monitored for about 4–5 months using the Dexcom G4 sensor, for which the sampling time is 5 min. Basal insulin was automatically recorded by the insulin pump (Roche Accu-Check Spirit Combo). Meal amount and timing were manually input in the system for all the meals. Based on this information, the system computed a suitable bolus of insulin. The data of each subject were split into a test set (about the 10% of the dataset), consisting of the last 10 monitoring days, while the remaining part was used as the training set (about the remaining 90%). In this dataset, an individual was discarded since more than 50% of the CGM trace was composed by missing values.

Table 1 and Table 2 report, for the OhioT1DM and CTR3 datasets, respectively, the percentage of missing values, the percentage of time spent in hypoglycemia (TBR), in target (TIR), in hyperglycemia (TAR), and the glycemic variability index [33] computed as $CV=100\cdot \frac{\sigma }{\mu }$, where CV is the coefficient of variation, σ is the standard deviation, and μ is the mean of the glucose levels.

Both datasets were acquired in free-living conditions, and they show a real-life scenario characterized by complex glucose dynamics, making the prediction of future glucose levels a challenging task. In the training set of both datasets, CGM gaps smaller than 30 min were filled using linear interpolation, while no imputation was performed on the test set. Looking at Table 1 and Table 2, a main difference among the two analyzed datasets can be found in the mean TIR: 66.4% vs. 78.4%, and in the mean TAR: 31% vs. 20%, for OhioT1DM and CTR3, respectively. This was partially expected since the CTR3 dataset is a closed-loop dataset; however, the mean CV, which is used to quantify the glycemic variability, is quite similar: 36.4% vs. 33%. In the following sections, the main steps of the proposed approach are described. Of note, the C-SARIMA, as described in [27], is designed to be tailored to individuals. Consequently, the following steps were computed for each individual of the dataset.

### 2.2. Time Series Segmentation

The first step of the methodology requires partitioning the CGM time series into a set of periods. To do so, exploiting the mealtime information, the postprandial period (PP) is defined as the CGM measurements:

- From mealtime up to 4 h after meal intake o;r
- From mealtime up to the following meal intake (if this happens before 4 h).

PPs containing more than one hour and a half (18 CGM samples) of missing glucose concentrations were discarded. Partitioning CGM time series in such a way leads to PPs having different lengths. To deal with this issue, PPs smaller than 4 h of monitoring data were expanded with blank values, i.e., not-a-number (NaN) values, to reach the maximum length. As a result, each CGM time series in segments had the same length. This is crucial for enforcing the seasonality and applying the methodology. After the NaN-padding step, a large number of PPs showed blank values in the final positions, and that should be adequately treated as missing data in the following steps.

### 2.3. Time Series Clustering

This step aims to group PPs that show a similar glycemic pattern. Following previous works, the partial distance strategy fuzzy C-means clustering (PDSFCM) was applied, since it can handle missing data, thus proving adequate for dealing with NaN-padded PPs and with incomplete data acquisitions. This clustering method is a modified version of fuzzy C-means (FCM) [34], which allows each PP to be included in several clusters with different degrees of membership. In particular, $w_{ij}$ denotes the degree of membership to the *i*-th cluster of the *j*-th PP. The degree of membership is a number in the range [0, 1], and the sum of the degrees of membership of each PP is 1:

(1) $$0\leq w_{ij}\leq 1\;and\;\sum_{i=1}^{nC}w_{ij}=1\forall j$$

PDSFCM finds the degree of membership for each PP in the clusters [34] by minimizing the following objective function:

(2) $$\sum_{i=1}^{nC}\sum_{j=1}^{N}w_{ij}^{m}d^{2}\left(x_{j},v_{i}\right)$$

where $x_{1},x_{2},\ldots ,x_{N}$ denotes the vector of the PPs’ glucose profiles; *N* is the total number of PPs; nC is the number of clusters (nC>1); *m* is the fuzzy exponent, i.e., a real number greater than 1; $v_{1},v_{2},\ldots ,v_{nC}$ are the cluster centroids defined as:

(3) $$v_{i}=\frac{\sum_{j=1}^{N}w_{ij}^{m}x_{j}}{\sum_{j=1}^{N}w_{ij}^{m}},1\leq i\leq nC$$

From now on, the center of the cluster (or cluster centroid) will be referred to as the cluster prototype.

Finally, $d(x_{j},v_{i})$ is the partial distance (i.e., a modified version of the Euclidean distance for dealing with missing values [35]) between any PP ($x_{j}$) and the cluster prototype *i*, $\left(v_{i}\right)$.

Given a set of centroids, $w_{ij}$ is computed using the following equation:

(4) $$w_{ij}=\frac{1}{\sum_{k=1}^{nC}{\left(\frac{d^{2}\left(x_{j},v_{i}\right)}{d^{2}\left(x_{j},v_{k}\right)}\right)}^{\frac{1}{m-1}}},1\leq i\leq nC,1\leq j\leq N$$

To compute the $w_{ij}$ minimizing (2), the centroid definition (3) and the membership Equation (4) are iteratively updated until no further improvement in the cost function is achieved.

Finding the right number of clusters is a critical task: a small number may result in clusters that are not completely separated; on the contrary, a large number may deteriorate the compactness of one or more clusters. For such a scope, many validation criteria have been proposed [34]. In this work, the optimal number of clusters nC, as well as the fuzzy exponent *m* were automatically chosen by minimizing the Fukuyama–Sugeno index [34,36] on the training set using an exhaustive grid search approach (ranges for nC = {2, …, 30} and for *m* = {1, …, 3}). Such an index measures both the compactness and the separation between each cluster and the prototypes.

### 2.4. Model Identification

Once the clustering step has been performed, several sets of “similar” glycemic profiles, having the same length, are obtained. Then, for each cluster, PPs are concatenated to obtain an artificial glucose time series, which shows an artificially induced seasonal pattern associated with the periodic meal consumption. By doing so, the seasonality, which is not originally present in raw CGM time series, is now enforced. Capturing the dynamics and the seasonality of the artificial concatenated time series can be performed by identifying a seasonal autoregressive integrated moving average (SARIMA) model for each cluster. A SARIMA model is a generalization of an autoregressive integrated moving average (ARIMA) model, which is able to take into account the seasonality. In fact, an ARIMA model can be described as follows:

(5) $$y\left(t\right)=\alpha +\omega \left(t\right)$$

(6) $$\phi_{p}\left(z^{-1}\right){▿}^{d}\omega \left(t\right)=\theta_{q}\left(z^{-1}\right)\epsilon \left(t\right)$$

where $y\left(t\right)$ is the CGM value at time *t*, α is the intercept, ω(t) is the disturbance series, ▿ is the backward differencing operator such that $▿\omega \left(t\right)=\omega \left(t\right)-\omega \left(t-1\right)$, and *d* is the order of the differencing step. ϵ(t) is a white noise process driving the model, and $\phi_{p}\left(z^{-1}\right)$ and $\theta_{q}\left(z^{-1}\right)$ are the polynomials of order *p* and *q* for the autoregressive and moving average part of the model. Similarly, a SARIMA model can be described by adding the seasonal terms to Equation (6):

(7) $$y\left(t\right)=\alpha +\omega \left(t\right)$$

(8) $$\phi_{p}\left(z^{-1}\right)\Phi_{P}\left(z^{-S}\right){▿}_{s}^{D}{▿}^{d}\omega \left(t\right)=\theta_{q}\left(z^{-1}\right)\Theta_{Q}\left(z^{-S}\right)\epsilon \left(t\right)$$

where *S* indicates the seasonality, ${▿}_{S}\omega \left(t\right)=\omega \left(t\right)-\omega \left(t-S\right)$, and *D* is the order of the seasonal differencing step. $\Phi_{P}\left(z^{-S}\right)$ and $\Theta_{Q}\left(z^{-S}\right)$ are the polynomials of order *P* and *Q* for the seasonal autoregressive and seasonal moving average part of the model. The SARIMA degrees of freedom, i.e., the order of the autoregressive (AR), moving average (MA), integrated (I) seasonal and nonseasonal parts, are chosen by minimizing the Bayesian information criterion (BIC) using an exhaustive grid search approach. In particular, the ranges for p={1,…,4}, $q=\left\{0,\ldots ,4\right\},d=\left\{0,1\right\},P=\left\{1,\ldots ,3\right\},Q=\left\{0,\ldots ,3\right\}$, and D={0,1} were considered. Following [27], the seasonality term (S) equals 53 samples: 48 samples that are the length of the PP plus 5 CGM samples that precede mealtime, the so-called pre-samples, introduced for a proper model initialization.

### 2.5. Real-Time Glucose Forecasting

**Figure 1:**
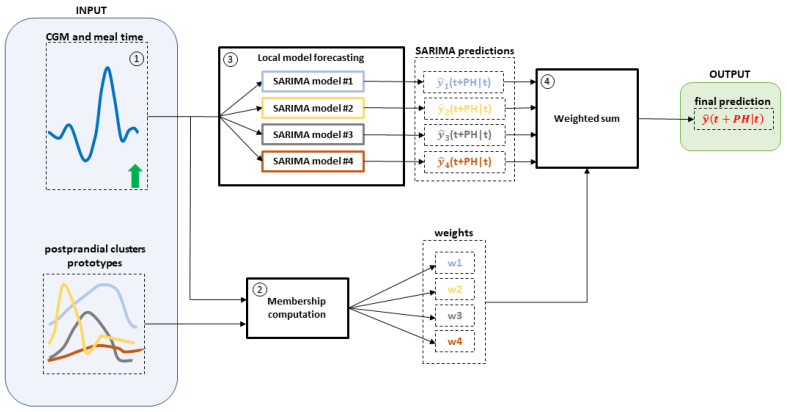
Schematic overview of the real-time prediction process. (1) CGM data (blue line) and mealtime (vertical green arrow), as well as postprandial clusters prototypes are the input of the forecasting process. (2) Postprandial cluster periods and CGM data after mealtime are used to compute the membership values (w1, w2, w3, w4). (3) CGM data and mealtime are fed into the SARIMA local models to provide the local predictions ${\hat{y}}_{1}\left(t+PH|t\right)$, ${\hat{y}}_{2}\left(t+PH|t\right)$, ${\hat{y}}_{3}\left(t+PH|t\right)$, ${\hat{y}}_{4}\left(t+PH|t\right)$. (4) Local predictions are then weighted according to the membership values to compute the final prediction. Each step is described in detail in Section 2.5.

Finally, once the SARIMA models are identified for each cluster, glucose can be predicted ahead in time by weighting the predictions of all SARIMA models. Figure 1 provides an overview of the forecasting process. As depicted in Figure 1, suppose that:

- The optimal number of clusters found in the training set is four (hence, four prototypes and four SARIMA models are available);
- It is mealtime (green vertical arrow in Figure 1).

The real-time glucose forecasting procedure is triggered at mealtime. The output is the predicted glucose level, indicated in Figure 1 as $\hat{y}\left(t+PH|t\right)$, and it can be computed by applying the following pipeline:

1. Wait for collecting 3 CGM samples (i.e., wait for 15 min, if the sampling time is 5 min);
2. Compute the membership values, i.e., the weights $\left(w_{1},w_{2},w_{3},w_{4}\right)$, between the collected CGM samples and the clusters prototypes using Equation (4);
3. Compute the glucose predictions exploiting the four identified SARIMA models (i.e., ${\hat{y}}_{1}\left(t+PH|t\right),{\hat{y}}_{2}\left(t+PH|t\right),{\hat{y}}_{3}\left(t+PH|t\right),{\hat{y}}_{4}\left(t+PH|t\right)$);
4. Compute the output $\hat{y}\left(t+PH|t\right)$ as the weighted sum of the computed predictions in Step 3 using the weights computed in Step 2;
5. Repeat Steps 2 to 4 each time a new sample is recorded.

As a final remark, the computationally demanding parts of the C-SARIMA are related to the clustering optimization procedure (i.e., determining the number of clusters and the fuzzy exponent) and to the local models’ identification process (i.e., SARIMA model order selection and parameters’ identification). However, these steps are computed only once and offline, leveraging training data. On the contrary, the online steps (described in Figure 1) are computationally inexpensive. In fact, each time a new CGM sample is recorded, the average time required to compute the PH step-ahead prediction was about 0.38 s, in detail: 32 μs for membership computation, 0.37 s for SARIMA models’ forecasting, and 9 μs for the weighted sum. The computation time was evaluated on an ASUS laptop equipped with an Intel (R) Core (TM) i7-8565U CPU @1.80 GHz 1.99 GHz.

### 2.6. Benchmark Glucose Predictive Algorithms

The effectiveness of the proposed approach based on clustering and SARIMA modeling was assessed by comparing the predicted PPs with the ones obtained by an individualized ARIMA model based on CGM data only and an individualized ARIMAX model fed by CGM, insulin, and CHO information. For each subject, an ARIMA and an ARIMAX model were identified. Similar to the SARIMA models, the order of the AR, MA, I, and exogenous (X) parts of the model were fixed for all subjects and chosen by minimizing BIC (among all the individuals) using an exhaustive grid search approach. In particular, the grid of explored order for AR = {1, …, 20}, MA = {0, …, 20}, I = {0, 1}, X = {1, …, 20}. Note that, while the model complexity was fixed, the model was individualized by estimating subject-specific model parameters. Finally, it could be of interest to investigate whether nonlinear models grant drastically different performances as compared to the proposed methodology. For such a scope, two feed-forward neural networks were considered as comparators. The first network (NN) is an effective state-of-the-art model for BG prediction [21], which employs CGM measurements up to 25 min before the current time as the input information. The second network (NN-X) is a variant of [21], which employs as the input: CGM readings, insulin, and CHO information. In both cases, the output is the glucose prediction PH minute-ahead in time. In detail, the NN and NN-X are composed of two hidden layers equipped with 10 and 5 neurons (with the sigmoidal transfer function) and an output layer equipped with a single neuron (with the linear transfer function). As concerns parameters’ learning (weights and bias), they are randomly initialized and updated according to a standard backpropagation training algorithm (Levenberg–Marquardt), which is applied in batch mode: weights and biases are updated when all the inputs and targets are presented. It is worth remarking that the training process must be performed for each PH.

### 2.7. Metric for the Assessment

The accuracy of the predicted PPs was evaluated for different PHs, i.e., PH = {30, 45, 60, 75} min. The root-mean-squared error (RMSE) between the predicted and the target CGM PP was considered:

(9) $$RMSE=\sqrt{\frac{1}{T}\sum_{t=1}^{T}{\left(y\left(t+PH\right)-\hat{y}\left(t+PH|t\right)\right)}^{2}}$$

where $y\left(t\right)$ is the current CGM reading, *T* is the length of the PP, and $\hat{y}\left(t+PH|t\right)$ is the PH step-ahead prediction using the information available up to time *t*.

## 3. Results

In this section, the performance of the proposed approach is presented. The novel approach is indicated as C-SARIMA in Table 3 and Table 4, with respect to the benchmark algorithms. All the algorithms were evaluated both on the OhioT1DM and CTR3 datasets.

Table 3 shows the results for OhioT1DM. Statistical significance was determined using a paired *t*-test if normality was accepted and a Wilcoxon signed-rank test if normality was rejected. The cross (+) indicates that there was a statistically significant difference (ssd) between the C-SARIMA and ARIMA. The asterisk (*) indicates that there was an ssd between the C-SARIMA and ARIMAX. The circumflex (^) indicates that there was an ssd between the C-SARIMA and NN. " indicates that there was an ssd between the C-SARIMA and NN-X.

At the short-term prediction horizon (i.e., ≤45 min), the proposed approach achieved similar performance to the individualized ARIMA model: there was no statistically significant difference among the two techniques. In particular, the RMSE provided by the proposed methodology was slightly higher (20.13 mg/dL vs. 19.64 mg/dL and 27.23 mg/dL vs. 26.91 mg/dL, for PH = 30, 45, respectively). However, for the long-term prediction horizon (i.e., ≥60 min), the performance of the C-SARIMA outperformed the ARIMA models (RMSE = 31.96 mg/dL vs. 33.67 mg/dL and 38.82 mg/dL vs. 33.91 mg/dL). In particular, for PH = 60 and 75 min, the difference was found to be statistically significant (*p*-values < 0.05). The NN performed similarly to the C-SARIMA (median RMSE of 20.11 mg/dL, 26.41 mg/dL and 32.11 mg/dL), and no statistically significant difference in the RMSE was found for PH ≤ 60 min. On the contrary, the C-SARIMA outperformed the NN for PH = 75 min by granting an RMSE = 33.91 mg/dL vs. 35.18 mg/dL (*p*-value < 0.05). Comparing the C-SARIMA with individualized ARIMAX models, it can be found that, for PH ≤ 45 min, the best results were obtained by individualized ARIMAX models (RMSE 18.73 mg/dL vs. 20.13 mg/dL and 26.46 mg/dL vs. 27.23 mg/dL). However, for PH = 60, 75 min, the C-SARIMA models provided results that did not differ in a statistically significant manner from the ARIMAX. Finally, the NN-X provided better results with respect to the C-SARIMA for PH ≤ 60 min: the RMSE was 17.78 mg/dL, 25.68 mg/dL, and 30.67 mg/dL, while no significant improvement was found for PH = 75 min (RMSE = 33.91 mg/dL vs. 34.06 mg/dL).

Table 4 shows the results for the CTR3 dataset. As for OhioT1DM, for short-term PH, the C-SARIMA provided similar performance to an individualized ARIMA, i.e., there was no significant improvement if compared to individualized ARIMA models: the median RMSE was 21.63 mg/dL vs. 21.02 mg/dL and 29.67 mg/dL vs. 29.42 mg/dL, for PH = 30 and PH = 45 min. However, for PH = 60 min and PH = 75 min, the proposed methodology outperformed the competitor, providing a statistically significant difference (median RMSE = 33.47 mg/dL vs. 35.38 mg/dL and 40.18 mg/dL vs. 44.01 mg/dL, respectively). The NN had performance comparable to the C-SARIMA for all the PHs ≤ 60 (median RMSE of 21.78 mg/dL, 30.64 mg/dL, 34.21 mg/dL) and inferior prediction for PH = 75 min (42.60 mg/dL vs. 40.18, *p*-value < 0.05).

A further assessment of the performance of algorithms using the same amount of information and an analysis of the performance when the size of the training set is varied are reported in Appendix A and Appendix B, respectively.

## 4. Discussion

The results among the two datasets were consistent: the proposed methodology based on clustering and the SARIMA models had comparable or superior performance with respect to one of the best-performing linear algorithms based on CGM data only, i.e., the individualized ARIMA model. In particular, the C-SARIMA outperformed the ARIMA for PH = 60 and 75 min. Furthermore, the results showed that the C-SARIMA was able to provide similar performance or slightly superior performance to a state-of-the-art nonlinear method for glucose prediction (NN). In particular, such a difference was found to be statistically significant for PH = 75 min.

The second linear comparator was an individualized ARIMAX model, which was expected to enhance prediction performance due to the use of additional information carried by insulin and CHO. In this comparison, the proposed approach provided performance that was not significantly different from the ARIMAX for PH = 45, 60, and 75 min. This is remarkable since the SARIMA and clustering-based approach use less information, CGM and mealtime only, while the ARIMAX also requires information about the CHO ingested and the amount of insulin administered, which represents a non-negligible drawback since the estimation of the correct amount of CHO and insulin is critical for subjects with T1D [37].

For the OhioT1DM dataset, a similar finding seems to hold also for the nonlinear comparator with inputs (NN-X). On the CTR3 dataset, no significant difference was found for PH = 60 min, whereas a significant (albeit hardly practically relevant) improvement was given by the NN-X with respect to the C-SARIMA for PH = 45 and 75 min. However, it is worth noting that on the OhioT1DM dataset, such an improvement was usually larger for short-term PH, but it became minor for long-term predictions.

When dealing with real data acquired in free-living conditions, the glucose response after meal intakes exhibits a wide range of variability. This variability forced the clustering step to use an increased number of clusters if compared to the results obtained on simulated datasets [27]. In fact, after the cluster optimization procedure, the mean number of clusters per subject was 16, while in [27], it was about 10. Being the first step of the pipeline, a successful clustering of the PPs is crucial for the success of the entire proposed methodology. In fact, if it provides several sets of “similar” glycemic responses, the resulting artificial seasonal CGM time series will show regular patterns periodically repeated. If this condition is satisfied, this leads to a better identification of SARIMA models and to an increased prediction accuracy.

**Figure 2:**
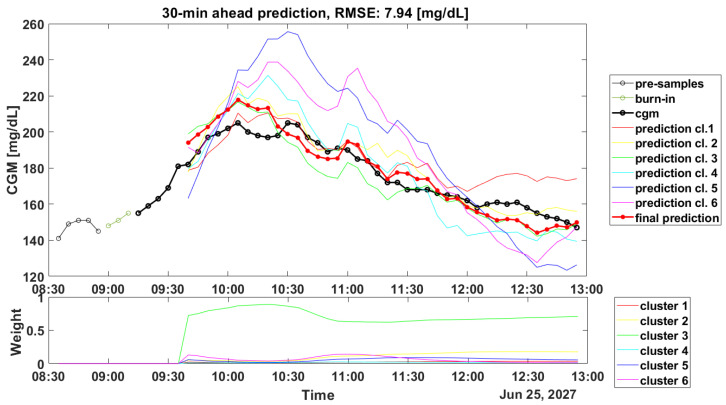
Illustrative example of a postprandial predicted profile, PH = 30 min. The top panel shows CGM data (black dotted line), the final prediction (red dotted line), and the predictions provided by each SARIMA model (colored lines). The bottom panel shows the prediction weights.

**Figure 3:**
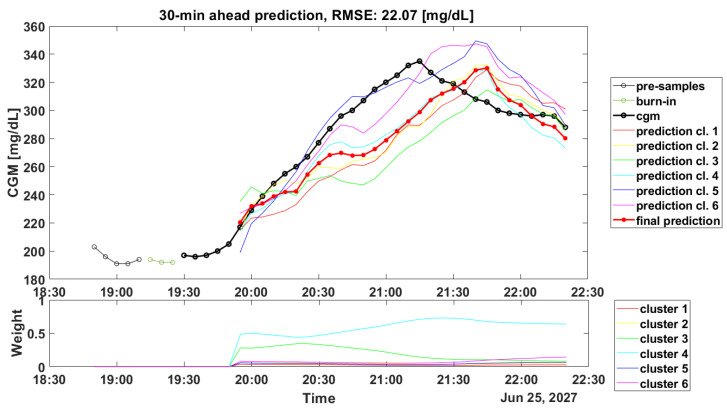
Illustrative example of postprandial predicted profile, PH = 30 min. The top panel shows CGM data (black dotted line), the final prediction (red dotted line), and the predictions provided by each SARIMA model (colored lines). The bottom panel shows the prediction weights.

Another critical aspect linked to the clustering step is about the computation of the weights during the real-time glucose forecasting. Such computation is crucial for obtaining accurate predicted profiles: in Figure 2 and Figure 3, the prediction results for a representative subject of the OhioT1DM dataset are shown (ID: 544), and it can be seen how the weights’ computation can lead to good and poor accuracy in the prediction of the PPs.

Figure 2 shows in the top panel the PP trace (black line) and the final prediction (red bold line). For a better visualization, 6 out of 12 predicted profiles (colored lines) were discarded since their weights (visible in the bottom panel) were almost equal to zero. Furthermore, in the top panel are also reported the 5 CGM samples (black thin line) before the meal (in this case, there is breakfast at 8.55) and the 3 CGM samples (indicated as burn-in in the legend) after the meal intake, which were used to compute the initial weights as described in the schematic overview of the forecasting process in Figure 1.

In Figure 2, the computed weights gave an accurate final prediction, since they assign the CGM data points to the most-similar cluster, in this case Cluster 3.

On the contrary, in Figure 3, which shows the CGM periods after dinner, the weights’ computation led to an incorrect assignment. Looking at the predicted profiles, it seems that the most-accurate predicted profile was the one obtained with the SARIMA model identified on Cluster 5 or on Cluster 6 (blue line and violet line, respectively). However, the highest weight was related to Cluster 4, which accurately forecast the initial samples (from 19.55 to 20.05), but then, it was not able to follow the target signal. Likely, the incorrect computation of the weights could be due to the fact that the prototypes in the training set were not completely able to describe the current PP, thus suggesting that a larger training set is required. Unfortunately, as shown in Table 4, similar results can be found even if a larger dataset, i.e., CTR3, is considered.

This work focused only on postprandial periods, since the proposed C-SARIMA algorithm is designed to provide effective prediction in these portions. Nevertheless, in a practical implementation, the algorithm can be easily extended to predict glucose levels over the entire time series, for instance by using a simple ARIMA model outside the postprandial window.

One important challenge in obtaining accurate BG predictions is the fact that the physiological response of T1D individuals varies over time, requiring the periodic update of the prediction algorithms. To address this problem in a practical implementation, the C-SARIMA could be modified by periodically repeating the proposed training pipeline (i.e., clustering step + SARIMA identification) on recent patient data. Notice that this update can be performed much less frequently than glucose prediction (e.g., once a week) and possibly on a remote server with massive computational resources. Alternatively, the real-time prediction algorithm update could be implemented by resorting to adaptive clustering algorithms [38] and adaptive SARIMA identification techniques [39].

Although the comparison with other literature works is not straightforward due to the fact that only the PPs and not the entire CGM traces are considered in this work, the numerical results seem in line with the results reported in [16,23,40,41,42]. In particular, the authors in [16] used a reduced version of the CTR3 dataset presented in this work, and their proposed method, which employed CHO and insulin as the input information, achieved a median RMSE = 31.7 mg/dL, for a 60 min PH, slightly better than that achieved by the C-SARIMA. Furthermore, the proposed methodology provides a performance similar to the one obtained by more complex deep learning methodologies exploiting additional information, as described in [43]. As a matter of fact, the C-SARIMA outperforms a multi-input and multi-step-ahead temporal convolutional network developed in [44], which provides RMSE = 23.22 mg/dL for 30 min PH. The authors in [45] employed a subset of the OhioT1DM dataset (only six subjects, corresponding to the 2018 release of the OhioT1DM dataset) and proposed a predictive algorithm based on stacked LSTM models and fed with more information than the C-SARIMA (i.e., meals, insulin, and step count). It is interesting to note that the results presented in [45], when no Kalman smoothing was applied to data, are comparable to the ones achieved by the proposed methodology (RMSE = 18.57 mg/dL, RMSE = 30.32 mg/dL, for PH = 30 min and PH = 60 min). In contrast, when the Kalman smoother was applied as a preprocessing step, their approach gave an RMSE = 6.45 mg/dL and 17.24 mg/dL for 30 min and 60 min PH. Unfortunately, these excellent prediction performances cannot be achieved in real-time, since the Kalman smoother proposed is non-causal. As such, it is not comparable to the methods investigated in this paper.

The C-SARIMA provides results that are in line even if compared to more complex models fed only by CGM data, such as the Echo State Network proposed by the authors in [46], which gave an RMSE = 21.7 mg/dL for 30 min PH. Instead, it should be noticed that our approach provides similar or slightly inferior performance if compared to algorithms that exploit physiological knowledge, as in [47], where the authors developed a patient-specific feed-forward neural network based on the transfer learning approach and integrated essential physiological knowledge into the structure, and as in [48], where the authors developed a predictive algorithms based on a simplified physiological model of glucose dynamics to generate features for a support vector regression computing the glucose prediction ahead in time. Such an approach granted an RMSE = 19.5 mg/dL and RMSE = 35.7 mg/dL.

Moreover, comparing the main findings with respect to previous works on this methodology shows quite different results in terms of performance metrics. In [26], the forecasting accuracy of the proposed methodology was measured by computing the RMSE and the MAPE for several PHs. Of note, the proposed methodology gave an RMSE = 9.99 mg/dL, 15.70 mg/dL, and 19.29 mg/dL for PH = 30, 45, and 60 min. However, the authors focused on evaluating how successfully the predicted trajectory fit the actual CGM data, which is different from evaluating the predicted glucose levels at a certain PH ahead in time, as described in [27] and in this work. Another limitation of [26] is related to the dataset: data were acquired during a clinical trial, which comprised 18 60 h closed-loop experiments based on scheduled meal intakes and exercise sessions. Due to the limited dataset, the reported results are related to the validation set only.

In the last work [27], the RMSE was computed as described in Equation (9), making a fair comparison between this work and [27] possible. In particular, the RMSE achieved by predicting postprandial periods and post-hypo treatment periods was about 15 mg/dL and 25 mg/dL for PH = 30 and 60 min, respectively. In this work, as shown in Table 3 and Table 4, the RMSE for PH = 30 and PH = 60 was about 21 mg/dL and 32 mg/dL. The main difference among these results can be found in the dataset: in [27], the authors exploited simulated datasets. These in silico simulations have been performed by exploiting a modified setup of the educational version of the UVA/Padova simulator [28]. In simulated datasets, glucose responses are quite similar and well defined: after meal intake, BG rises, and it comes back to the euglycemic range within 2.5 h from the meal.

## 5. Conclusions

In previous works, the C-SARIMA methodology for glucose forecasting, based on fuzzy C-means clustering and SARIMA models, was shown to outperform other literature methodologies, especially if long-term PHs are considered. However, the assessment of the methodology was limited to well-controlled simulated datasets, and a more robust validation on real and challenging dataset acquired in free-living condition was needed. In the present work, this assessment was performed by exploiting two datasets to take into account the different sizes of the datasets (i.e., the number of available monitoring weeks/months) and insulin administration regiments (manual control vs. hybrid closed loop). The results found on both datasets were consistent: the proposed C-SARIMA methodology outperformed individualized ARIMA models for PH > 45 min and the NN for PH > 60 min. Remarkably, there was no statistically significant difference between the results provided by the C-SARIMA and the ones provided by individualized ARIMAX models fed by CGM, CHO, and insulin information. Furthermore, it has been pointed out that the clustering step is crucial for obtaining sets of similar glycemic responses and for the computation of the weights in the forecasting process.

It should be stressed that the amount of input information can have a major practical impact on the applicability of the algorithms. In fact, insulin data cannot always be available in practice (including a large subpopulation of patients that used insulin pens instead of insulin pumps), and meal amount information can only be provided manually by the subject, after a cumbersome estimation procedure, which largely increases the therapy burden for the patient. In view of this, the proposed methodology represents an appealing option since it grants improved prediction performance with respect to methods using less input information without significant degradation with respect to methods using more input information.

## Figures and Tables

**Table 1:** Background information for the OhioT1DM dataset. Numerical values are rounded to the nearest integer.

Subj ID	Missing Values (%)	CV (%)	TIR (%)	TAR (%)	TBR (%)
540	8	41	72	22	6
544	15	36	70	29	1
552	23	37	80	18	3
559	11	42	61	36	4
563	7	33	73	25	2
570	5	33	43	56	2
575	7	42	70	23	7
584	8	35	53	46	1
588	3	30	63	37	1
591	12	37	68	28	4
596	18	34	78	20	2
Mean (SD)	11 (6)	36.4 (4)	66.4 (11)	31 (12)	3 (2)

**Table 2:** Background information for the CTR3 dataset. Numerical values are rounded to the nearest integer.

Subj ID	Missing Values (%)	CV (%)	TIR (%)	TAR (%)	TBR (%)
1	4	29	80	19	1
2	23	32	79	20	1
3	3	30	80	18	2
4	8	39	75	22	3
5	18	35	78	20	2
6	21	31	84	15	1
7	25	32	70	30	1
8	12	31	83	15	2
9	35	36	83	16	1
10	25	38	70	27	3
11	15	31	85	13	2
12	22	37	72	26	2
13	19	33	80	19	1
Mean (SD)	17.6 (9)	33.3 (3.4)	78.4 (5.1)	20 (5)	1.6 (0.8)

**Table 3:** Comparison of the performance of the C-SARIMA against the individualized ARIMA and ARIMAX models, and NN and NN-X on the OhioT1DM dataset.

Models	RMSE (mg/dL)			
	PH = 30 min	PH = 45 min	PH = 60 min	PH = 75 min
ARIMA	19.64	26.91	33.67	38.82
	[18.42–20.54]	[23.86–28.59]	[29.82–35.11]	[32.48–41.59]
NN	20.11	26.41	32.11	35.18
	[17.58–20.99]	[25.10–28.31]	[30.94–33.26]	[32.55–37.74]
C–SARIMA	20.13 (*,″)	27.23 (″)	31.96 (+)	33.91 (+,^)
	[18.63–21.38]	[24.63–28.74]	[29.55–33.95]	[31.97–37.29]
ARIMAX	18.73	26.46	30.82	34.73
	[17.31–20.06]	[22.96–27.03]	[29.30–31.92]	[31.31–39.09]
NN–X	17.78	25.68	30.67	34.06
	[16.79–21.04]	[24.85–27.62]	[28.98–34.93]	[32.71–35.54]

**Table 4:** Comparison of the performance of the C-SARIMA against individualized ARIMA and ARIMAX models, and NN and NN-X on the CTR3 dataset.

Models	RMSE (mg/dL)			
	PH = 30 min	PH = 45 min	PH = 60 min	PH = 75 min
ARIMA	21.02	29.42	35.38	44.01
	[20.03–24.86]	[27.40–33.24]	[34.63–40.48]	[39.50–45.86]
NN	21.78	30.64	34.21	42.60
	[19.35–24.23]	[26.88–34.11]	[29.92–38.68]	[35.97–44.42]
C–SARIMA	21.63	29.67 (″)	33.47 (+)	40.18 (+,^,″)
	[20.00–25.90]	[25.83–34.07]	[29.59–39.62]	[32.92–42.42]
ARIMAX	20.83	28.13	33.57	39.99
	[17.80–23.40]	[24.22–32.65]	[28.54–40.44]	[31.36–43.40]
NN–X	21.12	27.98	33.37	38.41
	[17.49–23.89]	[23.52–34.63]	[27.36–34.63]	[30.38–41.71]

**Figure A1:**
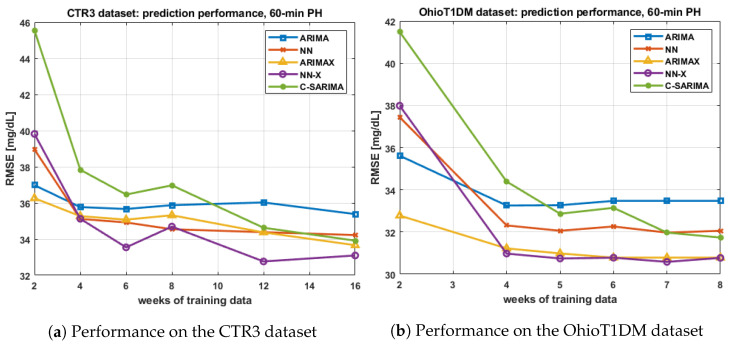
Predictive performance for increasing training set size. (**a**) Results on the CTR3 dataset; (**b**) results on the OhioT1DM dataset for the ARIMA (blue square), NN (orange cross), ARIMAX (yellow triangle), NN-X (violet circle), and C-SARIMA (green dots).

## Data Availability

Not applicable.

## Appendix A. Algorithms Employing the Same Amount of Information

Algorithms Employing the Same Amount of Information It could be interesting to investigate the performance of predictive models that employ the same information as the C-SARIMA. For such a scope, we propose ARIMAX+mealtime and NN+mealtime: two variants of the ARIMAX and NN-X fed by CGM and mealtime only. The results are reported in Table A1 and Table A2 for the OhioT1DM and CTR3 datasets, respectively. The asterisk (*) indicates if an ssd was found between the C-SARIMA and ARIMAX+mealtime, and (+) indicates if an ssd was found between the C-SARIMA and NN+mealtime.

**Table A1:** Comparison of the performance between the C-SARIMA vs. the individualized ARIMAX + mealtime and the NN + mealtime model fed by CGM and mealtime on OhioT1DM dataset.

Models	RMSE (mg/dL)			
	PH = 30 min	PH = 45 min	PH = 60 min	PH = 75 min
ARIMAX + mealtime	18.93	27.88	34.28	38.39
	[17.42–20.52]	[22.90–28.93]	[28.26–35.78]	[32.47–41.68]
NN + mealtime	20.16	26.53	32.78	34.22
	[18.04–21.88]	[23.06–28.28]	[30.55–33.88]	[31.36–37.81]
C–SARIMA	20.13	27.23	31.96 (*,+)	33.91 (*,+)
	[18.63–21.38]	[24.63–28.74]	[29.55–33.95]	[31.97–37.29]

**Table A2:** Comparison of the performance between the C-SARIMA vs. the individualized ARIMAX + mealtime and the NN + mealtime model fed by CGM and mealtime on the CTR3 dataset.

Models	RMSE (mg/dL)			
	PH = 30 min	PH = 45 min	PH = 60 min	PH = 75 min
ARIMAX + mealtime	20.97	29.40	36.75	42.95
	[17.83–24.63]	[23.36–33.36]	[29.90–41.74]	[36.35–44.44]
NN + mealtime	21.57	29.24	34.55	41.29
	[18.13–24.50]	[24.37–33.55]	[29.43–38.28]	[32.79–42.47]
C–SARIMA	21.63	29.67	33.47 (*,+)	40.18 (*,+)
	[20.00–25.90]	[25.83–34.07]	[29.59–39.62]	[32.92–42.42]

The numerical results were consistent between the two datasets: for PH = 30 min, the best results were achieved by the ARIMAX+mealtime (the median improvement was about 1 mg/dL on the OhioT1DM dataset and about 0.5 mg/dL on the CTR3 dataset with respect to the C-SARIMA). For PH = 45 min, the three methods achieved similar performance (about 27 mg/dL and 29 mg/dL, on the OhioT1DM and CTR3 datasets, respectively). As shown in Table A1 and Table A2, for PH > 45 min, the best results were achieved by the C-SARIMA, which outperformed its comparators (the improvement was statistically significant, *p*-value < 0.05). In fact, as detailed in Table A1, compared to the ARIMAX+mealtime and NN+mealtime, the C-SARIMA gave an RMSE = 31.96 mg/dL vs. 34.28 mg/dL vs. 32.78 mg/dL, for PH = 60 min, and an RMSE = 33.91 mg/dL vs. 38.39 mg/dL vs. 34.22 mg/dL, for PH = 75 min. Similarly, as shown in Table A2, compared to the ARIMAX+mealtime and NN+mealtime, the C-SARIMA gave an RMSE = 33.47 mg/dL vs. 36.75 mg/dL vs. 34.55 mg/dL, for PH = 60 min, and an RMSE = 40.18 mg/dL vs. 42.95 mg/dL vs. 41.29 mg/dL, for PH = 75 min.

## Appendix B. Performance of the Algorithms for Different Training Set Sizes

Figure A1 reports the prediction performance at PH = 60 min of the ARIMA, NN, C-SARIMA, ARIMAX, and NN-X, which were trained on a training set of increased size (from 2 to 16 weeks and from 2 to 8 weeks for the CTR3 dataset and OhioT1DM, respectively). As expected, the larger the training set size, the lower the RMSE was, up to a point, at which a further increase in the training data did not lead to an improved prediction. Furthermore, Figure A1 shows that less-complex models, such as the ARIMA and ARIMAX, achieved a plateau more rapidly (approximately after 2/4 weeks) than their nonlinear counterparts the NN and NN-X. The C-SARIMA showed the slowest convergence to the plateau level. Furthermore, it is interesting to note that, when the size of the training set was larger than 6 weeks, the RMSE for the ARIMA, NN, ARIMAX, and NN-X reached a plateau value (ranging from 32 to 35 mg/dL for the CTR3 dataset and from 31 mg/dL to 34 mg/dL for the OhioT1DM dataset). On the contrary, the C-SARIMA showed a decreasing trend in the RMSE, which did not reach a plateau with all the data available for training. In fact, this approach requires an adequate number of postprandial responses to create meaningful clusters and presents more parameters to estimate.

Based on Figure A1, one can conclude that the availability of a much larger dataset can be beneficial to further improve the C-SARIMA. However, we should stress that an individual affected by type 1 diabetes is subject to slow physiological changes, altering his/her response and requiring model update/re-identification on recent data. As a consequence, too old data can be hardly useful, if not detrimental. Once much larger datasets are available, future work will better address this open point.
